# Clinical and epidemiological aspects of patients with cow's milk allergy followed in a public health reference center in central Brazil

**DOI:** 10.1186/1939-4551-8-S1-A73

**Published:** 2015-04-08

**Authors:** Germana Pimentel Stefani, João Bosco Siqueira, Ludimila Porto L Nunes, Isabela De Oliveira Lima, Claudia Cantelli Daud Bordin

**Affiliations:** 1 Secretaria Municipal De Saúde De Goiânia, Brazil; 2 Federal University of Goias, Brazil

## Background

The prevalence of cow's milk allergy (CMA) has increased in last decades. The disease affects especially young infants and their clinical presentation can be diverse, often associated with comorbidities. The diagnosis is essentially based on clinical aspects and challenge tests. Its treatment demands strict adherence to elimination diet, with avoidance of milk protein and, for infants, the use of special hypoallergenic formulas. The increase in demand for these formulas, costly to the Public Health System, makes it necessary to better characterize the target population of assistance programs.

## Methods

This is a descriptive, observational, cross-sectional study based on secondary data collected from a standardized first consultation's form, regarding clinical and epidemiological aspects of patients who have requested special formulas due to previous diagnosis of cow’s milk allergy and were referred to a Municipal Health Centre of Food Allergy in Central Brazil.

## Results

Between January 2011 and May 2012, 449 patients (55 % male) were admitted. The mean age at admission was 11.34 months (± 8.03), while the average age of onset of symptoms suggestive of food allergy was 3.87 months (± 4.19). Almost 90% of the patients presented symptoms during the first year of life. Extensively hydrolyzed formulas were the mostly requested. Absolute predominance of not-IgE-mediated cases was noted (88,4%), typically with late presentation and preponderance of digestive (91.3 %) and systemic (33.4 %) symptoms, with rare anaphylactic reactions (03 cases). Children under one year have had significantly more digestive symptoms than older infants. At admission time, 24.6% infants younger than 6 months were underweight ( z < -2 weight-for-age score). Despite the family history of atopy in up to 75% of cases, over 80% of children had consumed milk with intact cow's milk protein at weaning. Half the patients using soy formulas have started their consumption before 6 months of life. The proportion of infants with the diagnosis of Gastroesophageal Reflux Disease was 68%, and 90% of them were taking acid suppressors drugs.

## Conclusions

The better characterization of the population seeking special formulas allows the generation of public health interventions in the fields of prevention, diagnosis and treatment, and also optimize overall patient care combined with effectiveness and lower costs.

